# No-cost meals might not exist for insects feeding on toxic plants

**DOI:** 10.1242/bio.059800

**Published:** 2023-06-19

**Authors:** Prayan Pokharel

**Affiliations:** Department of Applied Entomology, Institute of Phytomedicine, University of Hohenheim, Otto-Sander-Straße 5, Stuttgart, 70599 Germany

**Keywords:** Plant toxins, Detoxification/sequestration, Chemical defence, Ecophysiological framework, Trade-offs, Cost and benefits

## Abstract

Plants produce chemicals (or plant specialised/secondary metabolites, PSM) to protect themselves against various biological antagonists. Herbivorous insects use plants in two ways: as a food source and as a defence source. Insects can detoxify and sequester PSMs in their bodies as a defence mechanism against predators and pathogens. Here, I review the literature on the cost of PSM detoxification and sequestration in insects. I argue that no-cost meals might not exist for insects feeding on toxic plants and suggest that potential costs could be detected in an ecophysiological framework.

## Teaser

**Summary:** Detection of physiological and ecological costs of chemical defence in insects under an ecophysiological framework for the better understanding of the ecology and evolution of plant-insect-antagonist interaction.

## Introduction

Predators are constant agents of natural selection that regulate ecosystems and sustain biodiversity on local and global scales. Predation risk affects the evolution of prey species through unique evolutionary processes that offer protection against predators. In general, insects protect themselves from their natural antagonists in several ways, including chemical, physical, morphological, or behavioural, although they are not exclusive to each other. For example, some insects use chemical defensive toxins (Eisner et al., 2005; Pokharel et al., 2020), hair (Sugiura and Yamazaki, 2014), and spines (Ito et al., 2016; Murphy et al., 2009). As defensive warning signals, many insects masquerade and mimic using cryptic and/or aposematic colours and patterns (Ruxton et al., 2019). Furthermore, numerous insects exhibit defensive behaviours, such as death-feigning (Humphreys and Ruxton, 2018), kicking (Catania, 2018; Gnatzy and Heusslein, 1986), stinging (Schmidt, 2016), and producing sounds (Bura et al., 2016).

Herbivorous insects have evolved various tolerance and resistance strategies to overcome plants' defences known as plant secondary/specialised metabolites (PSM). Furthermore, insects can accumulate PSMs while feeding on plants, using them for defence against biological antagonists, a common phenomenon observed in more than 275 insect species called sequestration, a syndrome of selective uptake, transport, modification, storage, and deployment of PSMs as a chemical defence (Beran and Petschenka, 2022). In contrast, many insects can synthesise their chemical defences, as observed in chrysomelid beetles (Pasteels et al., 1988) and *Heliconius* spp. butterflies (Brown and Francini, 1990). However, toxins are sequestered in tissues and have a physiological impact on internal targets. For example, cardenolide-sequestering insects of at least seven different orders have evolved target site insensitivity (TSI) in Na^+^/K^+^-ATPases (a ubiquitous animal enzyme that drives various essential physiological functions) where cardenolides bind (Dobler et al., 2012; Karageorgi et al., 2019). TSI is also observed in other specific receptors (for example sodium channels, neurotransmitters, and microtubules) for toxins, such as pyrethrins and alkaloids (Wink, 2009). Furthermore, some plants possess two-component defences as inactive glycosylated storage forms, which must be activated by β-glucosidases from the plant, insect, or both. In this category, several insects sequester toxins, including benzoxazinoids, cyanogenic glycosides, glucosinolates, iridoid glycosides, and salicinoids (Opitz and Müller, 2009). Nevertheless, defensive chemicals are highly complex mixtures that can be acquired from the environment or biosynthesised, which are widespread and exhibit high levels of variation even within an insect species (Blum, 2012). The answer to the critical question of whether insect individuals pay a price for chemical defence remains unresolved, and this Review focuses solely on the potential underlying costs of insect defensive chemicals.

**Fig. 1.**
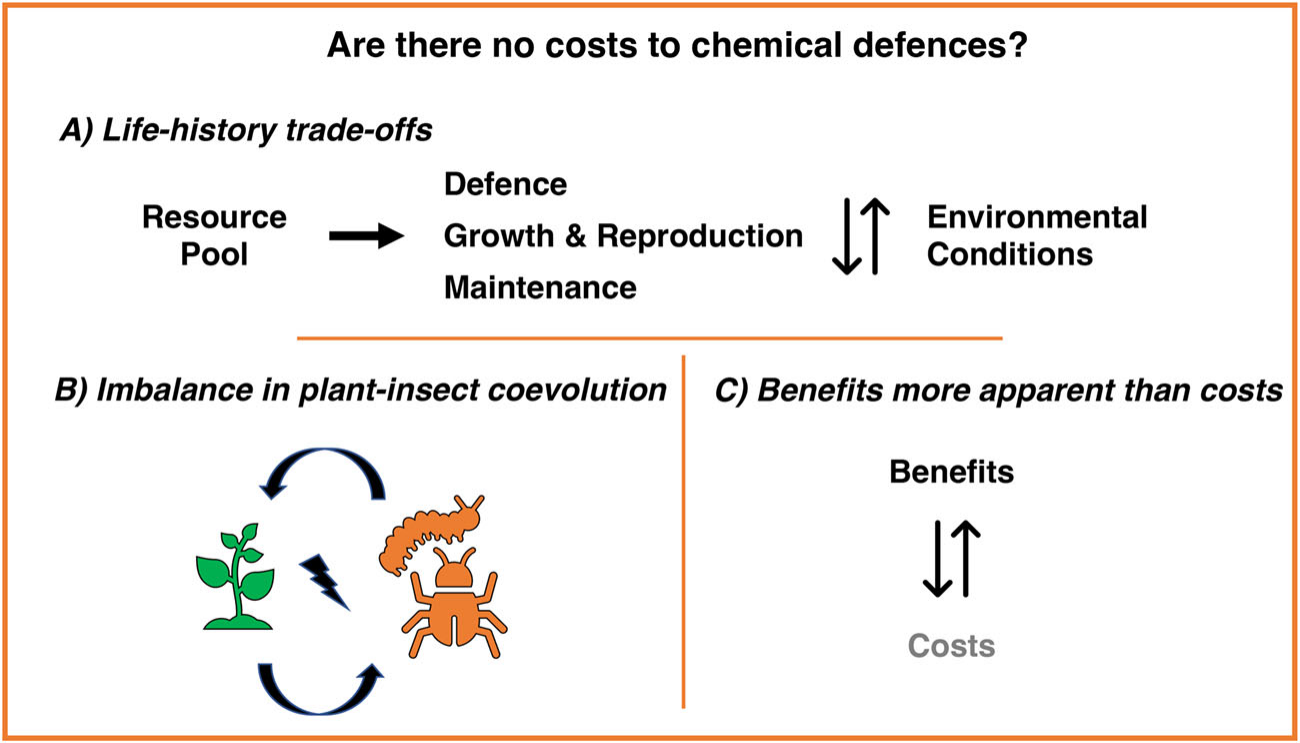
Consequences and implications of no-cost to chemical defence.

If an insect produces defensive compounds, there are costs associated with expressing the requisite enzymes for toxin synthesis, storing toxins, and avoiding autotoxicity. Some of these costs are avoided in sequestration by deriving PSMs directly from host plants, but other related costs remain, such as toxin transport through the gut and metabolism (biotransformation). In addition to these costs, there is also the cost of resistance to toxins, usually linked to the specific molecular mechanisms of resistance. Despite its relevance, little is known about the cost-benefit analysis of various processes involved in chemical defence. It is assumed that there is minimal or no cost for chemical defences (Zvereva and Kozlov, 2016). Alternatively, costs are not always easy to detect or estimate (Lindstedt et al., 2010; Ruxton, 2014). Therefore, it is important to ask: 1) Do chemical defences come free (or at a very low cost)? 2) Is it particularly challenging to identify the cost of chemical defences (Fig. 1)?

To begin, a very weak or no cost of defence rattles the central and recurring theme in ecology and evolution: the life history trade-off (Fig. 1A). Life history theory focuses on how evolution, a natural selection imposed by ecological challenges, drives organisms to maximise their fitness (Roff, 1992; Stearns, 1992). Fitness is the sum of the reproductive success of an organism, and life history traits are the main fitness parameters. Investments in physiological and ecological traits such as defence, maintenance, growth, and reproduction cause trade-offs owing to the restriction imposed by genetic, developmental, physiological, and phylogenetic limits. Therefore, ecophysiological trade-offs are caused by competitive resource allocation, i.e. resources invested in one trait cannot be invested into another (Stearns, 1992). Each trait is anticipated to manifest differently depending on environmental conditions (Clark and Harvell, 1992). In this context, significant empirical evidence and several theoretical models have been presented (Houston et al., 1993; McPeek, 2004; Steiner and Pfeiffer, 2007; Stoks, 2001). Optimal time and resource allocation strategies differ in various situations, such as benign environmental situations (low predator densities and high resources) and harsh environmental situations (high predator densities and low resources).

Many theories have been formulated to understand how interacting species affect each other's evolution (Abrams, 2000; Thompson, 2005). Most importantly, theoretical models of victim-exploiter relationships (such as plant-insect, prey-predator, and host-pathogen interactions) assume the costs of exploiters' adaptations to overcome victims' defences (Bergelson et al., 2001). Plants have a cost for the biosynthesis, storage, and maintenance of PSMs (Koricheva, 2002; Neilson et al., 2013). Regardless of their deployment in constitutive or inducible defences, plants need to invest in PSMs. Zero investment by insect herbivores in resistance and tolerance to PSMs establishes a coevolutionary asymmetry between plant and insect interactions (Fig. 1B). Furthermore, the evolutionary arms race between plants and insects (in terms of PSM sequestration) is not an evolutionary dead end but rather a driver of the escalation of symmetrical coevolution (Petschenka and Agrawal, 2016).

The evolution of chemical defences is considered in association with the trade-offs between acquired benefits through protection against natural enemies and the possible defence costs. The costs of possessing chemical defences are assumed to be compensated by increased protection against predators and parasites (Bowers, 1992; Camara, 1997). In other words, the costs of chemical defences are often outweighed by their benefits (Fig. 1C) and such costs are not always easy to detect and estimate (Lindstedt et al., 2010). The complications in detecting and estimating the costs may be due to experimental designs that cannot control for all factors and manipulate only one variable (Stearns, 1992). Furthermore, natural selection may favour cost-effective traits, making it challenging to detect costs (Fry, 2003).

## Costs of chemical defence in an ecophysiological framework

**Fig. 2.**
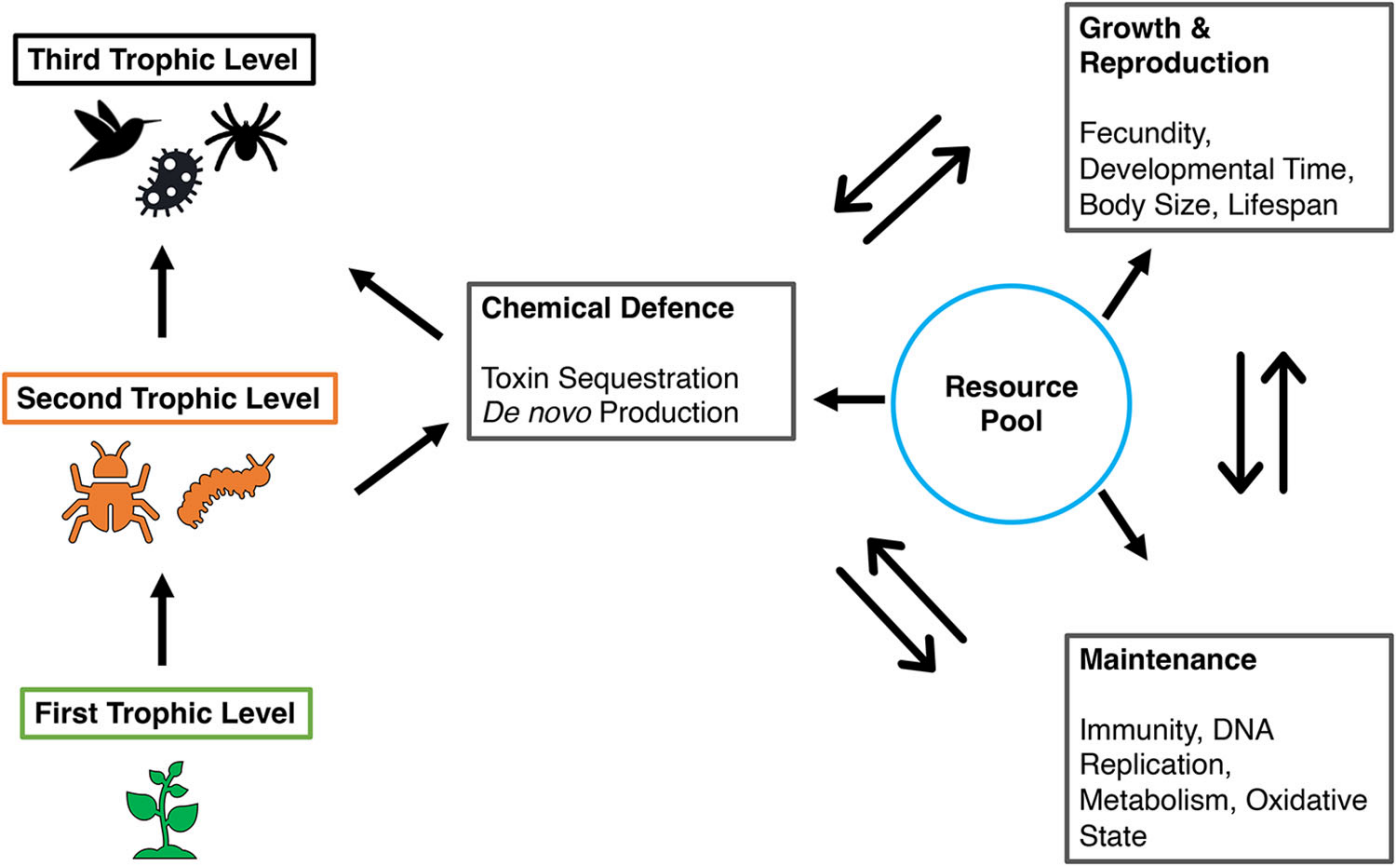
**Chemical defence in an ecophysiological framework.** Organisms have a finite pool of resources that must be allocated to diverse functions such as growth, reproduction, maintenance, and defence. The two-headed arrows signify trade-offs that arise from the allocation patterns of the resource pool.

Chemical defences can be acquired through sequestration, *de novo* biosynthesis, or a hybrid of these two strategies. Sequestration is the phenomenon of toxin uptake from host plants (i.e. first trophic level) by insects (i.e. second trophic level) to protect themselves against predators and parasites (i.e. third trophic level). Chemical defence is often associated with warning colours and patterns (called aposematism), and sequestration and aposematism are the prominent suites of defences in many species. In an ecophysiological framework, the costs of chemical defences are expected to be seen in many aspects, including oxidative stress, reduced immune defence, impaired growth, and reduction in fecundity (Fig. 2).

## Physiological costs

### Immunological costs

Recent literature shows that the immune response (Smilanich and Muchoney, 2022) and toxin sequestration (Beran and Petschenka, 2022) are two of the vital physiological links between plant-insect-antagonist (here, biological antagonists such as pathogens and predators) interactions. Empirical studies have shown that some toxin-sequestering insects feeding on toxic host plants are protected against pathogens. However, this may be costly. Some insects compromise their immune systems while feeding on toxic dietary resources (Lampert, 2012; Smilanich et al., 2009). Although in some cases toxin-sequestering insects have a weak immune system, they are less likely to be deterred by predators as they are laden with toxins. If this is the case, then the insect host is vulnerable to parasitisation due to its weak immune system. However, the insect host would be a safe haven for parasites. Therefore, both the host and the parasite are protected (Gentry and Dyer, 2002; Harvey et al., 2005; Reudler et al., 2011).

Increasing amounts of iridoid glycosides (IG) sequestered by Buckeye caterpillars *Junonia coenia* have been linked to a compromised immune response (Camara, 1997; Smilanich et al., 2009). A negative effect on the immune response of *J. coenia* caterpillars was observed when they were fed diets containing higher IG levels and sequestered these toxins compared to caterpillars fed diets containing lower IG (Smilanich et al., 2009). In addition to laboratory studies, a negative association was observed between IG sequestration and the immune response in the Baltimore checkerspot caterpillar *Euphydryas phaeton* in a field-based study, implying that toxin sequestration suppresses cellular immune responses (Muchoney et al., 2022).

Contrary to these findings, in *Grammia incorrupta*, the concentration of IGs did not affect immune responses, when caterpillars were fed IG-containing plants (Smilanich et al., 2011). Similarly, the amount of cardenolides present in different host plants did not affect the immune response of cardenolide-sequestering monarch butterflies *Danaus plexippus* (Adams et al., 2021). Furthermore, gene expression analysis demonstrated that monarch butterflies did not show differential immune responses in monarch butterflies to the common protozoan parasite *Ophryocystis elektroscirrha* infection (Tan et al., 2019). However, feeding on milkweed plants containing large amounts of cardenolides reduced *O. elektroscirrha* infection and growth in monarchs (Gowler et al., 2015; Sternberg et al., 2012; Tan et al., 2018). Interestingly, withanolides had a positive effect on specialist *Heliothis subflexa* larvae that fed on *Physalis* spp. by increasing larval growth and immune system activity, but this positive effect was not observed in the closely related *H*. *virescens* (Barthel et al., 2016). Enzymes such as phenoloxidase (Liu et al., 2009) and glutathione (McMillan et al., 2018) are important for both immune functions and toxin detoxification. In an ecophysiological framework, the costs of chemical defence may exist in a competing energy demand between sequestration and immunity, which differs among insect groups sequestering different classes of PSMs.

The discrepancy in the response to immunity could also be due to the different experimental methods used to measure the immunity. In summary, insects have innate immune systems composed of cellular and humoral components. The cellular components include phagocytes, which engulf and destroy invading pathogens, and haemocytes, which are specialised immune cells that circulate throughout the body of the insect. The humoral components include antimicrobial peptides, which can kill pathogens, and the melanisation response, which involves the formation of a melanin pigment around invading microorganisms to keep them isolated from the body. Insects can be negatively influenced by a variety of factors, such as poor nutrition, exposure to extreme temperatures, pesticides, pollutants, and other environmental toxins, which can make them susceptible to infection. In addition, some parasites and pathogens have evolved mechanisms to suppress or evade the insect immune system (Beckage, 2011). Therefore, to conclusively establish an immune response, multiple methods that measure both cell-mediated and humoral immunity should be performed in tandem (Adamo, 2004). A cell-mediated immune response can be tested by melanisation [using dextran beads and nylon filament (Laurentz et al., 2012; Smilanich et al., 2009)] and haemocyte attributes [such as enlargement, agglutination, denucleation, shape distortion, and abnormal staining of the cells (Muchoney et al., 2022; Perveen and Ahmad, 2017)]. Similarly, a humoral immune response can be estimated using biochemical assays [phenoloxidase activity (Barthel et al., 2016), relative protein concentration (Rahman et al., 2004), lysozyme-like activity (Charles and Killian, 2015)] and zone of inhibition assays [using lipopolysaccharide (a cell wall component of bacteria) in the form of antimicrobial growth (Adams et al., 2021)]. Therefore, for assessing the immune response, the best would be to combine the methods such as bioassays, genetic analyses, microscopy, and molecular techniques.

### Metabolic costs

Some insects such as *Heliconius* species have evolved to biosynthesise cyanogenic glucosides, while they already possess mechanisms to tolerate autotoxicity and have evolved to sequester from their host plant *Passiflora* spp. to reduce the energetic costs of biosynthesis (Zagrobelny et al., 2008). Empirical evidence regarding the metabolic costs of toxin sequestration is scarce. For example, the respiration rate (i.e. CO_2_ production) of *J. coenia* caterpillars was negatively correlated with catalpol (an IG) present in their diet (Smilanich et al., 2009). Similarly, in *Ceratomia catalpae* sphinx caterpillars, respiration rate was negatively correlated with the amount of sequestered catalpol (Lampert, 2020). It is important to note that the respiration rate and catalpol concentration per dry mass of caterpillars were positively correlated (Lampert, 2020). Recent evidence suggests that cardenolide sequestration is costly and affects the flight energetics of monarch butterflies (Pocius et al., 2022). We can speculate that in some insect species, the resource pool is allocated according to metabolic (energetic) demands, which is also observed beyond insects (Box 1).

**Box 1. Costs of chemical defence beyond insects**

Beyond insects, literature suggests that the production and maintenance of chemical defence is metabolically costly. For example, viper snakes needed more than 28 days to fully regenerate their venom (Oron and Bdolah, 1973; Rotenberg et al., 1971), whereas tarantula spiders needed up to 85 days (Perret, 1977). During the first 72 h of venom regeneration, the metabolic rate increased by 11% in the pit viper snakes (McCue, 2006) and by 40% in the scorpion *Parabuthus transvaalicus* (Nisani et al., 2007). Furthermore, the garter snake *Thamnophis sirtalis* evolved tetrodotoxin (TTX) resistance as a result of arms-race coevolution with their toxic newts *Taricha* spp. Toxin-resistant snakes (with just four amino-acid substitutions) significantly reduced crawl speed, a measure of organismal performance (Hague et al., 2018). On the other hand, TTX production as a defence mechanism is also costly for newts (Hanifin, 2010; Mailho-Fontana et al., 2019). Evidence suggests that secondary loss of the venom system often occurs, implying a significant biochemical cost. Marbled sea snakes *Aipysurus edyouxii* have been reported to have lost their active venom due to a change in the dietary source (Li et al., 2005). Similarly, the cribellate orb weavers (Uloboridae), which kill their prey by encasing them firmly in silk, secondarily lost their venom (see review Correa-Garhwal et al., 2022).

Insect adaptations to host-plant toxins involve mechanisms that metabolise toxins and prevent autotoxicity (Agrawal et al., 2022). In cardenolide-sequestering *Aphis nerii*, gene expression analysis (Birnbaum et al., 2017) showed the expression of a variety of classical detoxification gene families, such as cytochrome P450s, UDP-glucuronosyltransferases (UGTs), and ATP-binding cassette transporters (Heckel, 2014), when the insects were fed on cardenolide-producing plants. Similarly, in *D. plexippus*, which sequesters cardenolides*,* transcriptome analysis showed upregulation of glutathione S-transferase (GST) and carboxyl esterase genes in the gut tissues of caterpillars fed plants with higher toxicity (Tan et al., 2019). It is very important to note that *A. nerii* and *D. plexippus* are cardenolide-sequestering specialists; however, *A. nerii* lacks cardenolide-resistant Na^+^/K^+^-ATPases (but see Karageorgi et al., 2019) and *D. plexippus* possesses cardenolide-resistant Na^+^/K^+^-ATPases that mediate resistance to cardenolides via target-site insensitivity (Zhen et al., 2012). Although possessing or not possessing the resistance mechanism to toxins, both insects were challenged by the presence of dietary toxins. Therefore, the above findings indicate that metabolic costs exist in sequestering species and warrant further investigation to test the possible crosstalk between toxins and specific genes that are directly involved in their modification and transport.

### Oxidative stress costs

When the capacity of the antioxidant defence and repair mechanisms of an organism is exceeded, oxidative stress is triggered by a rapid increase in the generation of reactive oxygen species (ROS) (Finkel and Holbrook, 2000; Metcalfe and Alonso-Alvarez, 2010; Monaghan et al., 2009). ROS are natural by-products of metabolic activities, and unless suppressed by antioxidants, they can damage a variety of biomolecules (such as DNA, proteins, and lipids) because of their instability and high reactivity (Balaban et al., 2005; Dowling and Simmons, 2009). In the early 1990s, Aucoin et al. published extensively on the oxidative stress in insect herbivores caused by phototoxins – toxic plant chemicals that are activated by the absorption of light (Aucoin et al., 1995, 1990, 1991). The impact of Anthropocene activities on insects has generated a large body of literature in which researchers have investigated different markers of oxidative stress associated with environmental pollution by heavy metals (Abdelfattah and El-Bassiony, 2022; Kafel et al., 2021; Renault et al., 2016) and pesticides (Hamama and Fergani, 2019; Palma Onetto et al., 2021). However, little is known about the possible oxidative stress in insect herbivores when dealing with plant toxins.

Theoretically, resistance and tolerance strategies to deal with toxins can damage molecules, such as lipids, proteins, and DNA, due to oxidative stress. Therefore, antioxidant defence systems must exist to combat ROS and repair or mitigate damage which is presumably very costly. Different levels of oxidative stress can occur and accumulate throughout an individual's lifespan (Beckman and Ames, 1998), which suggests that oxidative stress in organisms is a potential driver of life history trade-offs (Costantini, 2008; Dowling and Simmons, 2009; Isaksson et al., 2011; Metcalfe and Alonso-Alvarez, 2010; Monaghan et al., 2009; Speakman and Garratt, 2014). Although ROS are usually associated with oxidative stress and cellular damage, some studies have suggested that ROS can also possess antioxidant properties. For example, certain ROS, such as hydrogen peroxide, can act as signalling molecules that prompt protective responses within cells (Veal and Day, 2011). Low levels of ROS can stimulate the organism's antioxidant defences by producing enzymes such as superoxide dismutase and catalase, which can help neutralise excess ROS and prevent oxidative damage (Bhattacharya, 2015). While low levels of ROS may have beneficial effects, high levels can be damaging. The redox signalling hypothesis postulates that there is a trade-off between ROS production and ROS regulation. Both processes can alter vital physiological functions that influence life history traits in organisms (Costantini, 2019).

**Fig. 3.**
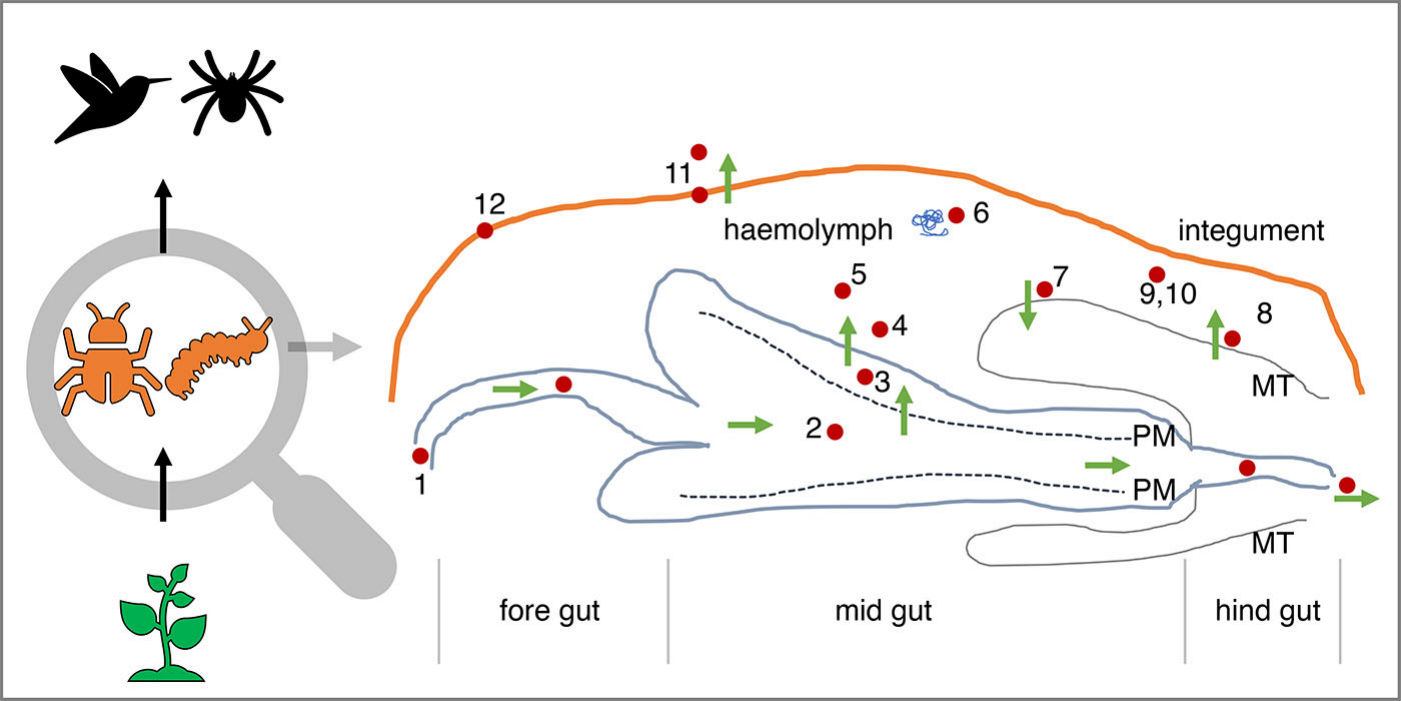
**Sites for the detection of costs of chemical defences.** The figure shows a schematic representation of a typical insect digestive system. An insect chews/sucks the PSM-rich food where various salivary enzymes (Musser et al., 2002) act together (1). The food is ingested and gets in contact with the peritrophic membrane (PM) (2). The PM is not just a physiological membrane, but also an extracellular matrix composed of chitin and glycoproteins lacking lipids. It protects the gut epithelium from oxidative stress by scavenging ROS, thereby preventing the exhaustion of other antioxidants (Summers and Felton, 1996). Furthermore, it functions as an immune barrier against pathogens (Kuraishi et al., 2011) (2). The PM separates the epithelial lumen into two partitions: the endoperitrophic space, which contains the food bolus, and the ectoperitrophic space, which contains the digestive enzymes and secretion products of the lumen. The digestive enzymes (3) can metabolically alter the PSMs (Ratzka et al., 2002) before they can enter into the haemocoel via diffusion and/or transport carrier (Kowalski et al., 2020; Strauss et al., 2013) (4). If PSMs cannot pass through, they are excreted by defaecation. Within the haemocoel (5), PSMs can be metabolised (Smagghe and Degheele, 1997); fat bodies (6) can also play a role in metabolization (Petersen et al., 2001). Some PSMs are excreted (Lindstedt et al., 2010) through the Malpighian tubules (MT) (7). In some cases, PSMs can be metabolised, reabsorbed and retained into hemocoel from MT (Chahine and O'Donnell, 2011; Reynolds et al., 2021; Yang et al., 2021) (8). Some PSMs can be transported to glands (9) (Hartmann, 2004) and vacuolated cells (10) (Scudder and Meredith, 1982). To release PSMs, the integument tissues can rupture (11) (Bramer et al., 2017). Furthermore, chemical defences are often linked with colourful warning signals (aka aposematism, 12) (Mappes et al., 2005).

Estimating the cost of oxidative stress is difficult, because an individual insect is often regarded as a uniform entity. In reality, an individual is a complex assembly of tissues/organs involved in distinct physiological roles with different characteristics, including the antioxidant system and cellular turnover (Costantini, 2019). Similarly, several physiological mechanisms are involved in dealing with toxins where costs can occur (Fig. 3). Insects exhibit tissue-specific antioxidant profiles. For example, the peritrophic matrix (a membrane that surrounds the food bolus and gut) can serve as a functional antioxidant (Summers and Felton, 1996), protecting the intestinal epithelium from oxidative damage caused by PSM ingestion (Barbehenn and Stannard, 2004). Interestingly, studies suggest that the oxidative radicals and antioxidant enzymes that deal with PSM are compartmentalised in the digestive tract, with higher amounts of superoxide anions and hydrogen peroxide in the foregut and higher enzyme activities of superoxide dismutase and catalase in the midgut (Krishnan and Sehnal, 2006).

Empirical studies on insects have shown that antioxidant molecules play a dual role in the detoxification of toxins (Enayati et al., 2005) and colour pigments (Shamim et al., 2014). Similarly, pigment molecules, including melanins, pterins, carotenoids, and flavonoids, can act as antioxidants (McGraw, 2005). Some insects, such as *Trichoplusia ni*, sequester carotenoids at concentrations up to 20 times higher than those found in their host plants (Nguyen et al., 2019). Carotenoids in invertebrates have a dual role; they can act as antioxidants and play a role in the expression and regulation of immune genes (see review Tan et al., 2020). Interestingly, a well-studied plant toxin, catalpol (an IG), which is sequestered by many caterpillars (see above), can act as an antioxidant (Bhattamisra et al., 2020). Recently, a study showed a physiological association in *D. plexippus* between oxidative stress (measured by oxidative lipid damage), cardenolide sequestration, and warning colouration (Blount et al., 2023). Interestingly, in *Oncopeltus fasciatus*, the amount of sequestered cardenolides depleted the redox state (measured by the total glutathione level) and antioxidant availability was traded off with brightness and chroma signals (Heyworth et al., 2023). Although very few studies have supported the physiological cost of toxin sequestration in aposematic insects, there is still a lack of evidence that toxin sequestration, warning signals (pigments), and oxidative stress are interlinked.

### Life history costs

Empirical evidence on the costs of chemical defences in life history (such as growth, fecundity, and lifespan) is equivocal, as different studies show contrasting outcomes. Cardenolide-sequestering insects grew faster with a larger adult body size when they were fed and raised on a cardenolide-rich diet (Blakley and Goodner, 1978; Chaplin and Chaplin, 1981; Isman, 1977; Krueger et al., 2021; Pokharel et al., 2021; Rothschild et al., 1975). Similarly, while feeding on plants producing higher concentrations of IGs, IG-sequestering caterpillars survived better, gained more weight, and developed faster into butterflies (Bowers and Puttick, 1988, 1989; Harvey et al., 2005; Saastamoinen et al., 2007). In contrast, insect species that sequester cardenolides did not show measurable differences in growth when fed plants containing a different amount of cardenolides (Erickson, 1973; Petschenka and Agrawal, 2015). However, recent research indicates that processing a highly potent cardenolide (vorusharin) into other cardenolides and sequestering them came at a cost in terms of growth for monarch butterflies (Agrawal et al., 2021).

Positive correlations between protective and nutritive phytochemical constituents, as well as the high degrees of adaptation in specialists to utilise plant compounds as nutrients, may be the cause of the non-appearance of costs in toxin sequestration. Interestingly, specialist insects such as *Pieris rapae* and arctiid moths (such as *Utetheisa* spp. and *Tyria* spp.) show a predilection for their host plants with glucosinolates (Renwick and Lopez, 1999) and pyrrolizidine alkaloids (Rothschild et al., 1979), respectively, which have been suggested to be addicted to plant chemistry (Wink, 2018). Specialists may suffer from the absence of dietary toxins due to selection for physiological homeostasis under continuous exposure to toxins. An ‘evolutionary addiction’ might be stated as the positive impact of toxins on the life history of specialist insects (Pokharel et al., 2021). Alternatively, several studies on pest insects have shown positive effects on fitness under insecticide stress, presumably due to hormetic effects (Celestino et al., 2014; Piiroinen et al., 2014). In terms of its functional rationale and potential fitness effects, insecticide-induced hormesis in arthropods is still puzzling, yet an emerging topic (Sebastiano et al., 2022).

### Ecological costs

It is commonly acknowledged that sequestered plant toxins effectively protect insects from both vertebrate (such as birds) and invertebrate (such as spiders) predators (Nishida, 2002). However, the literature suggests that the chemical defence of prey is not universal against all predators, implying additional ecological costs together with the associated physiological costs. For example, the sequestration of cardenolides by a milkweed bug *Lygaeus equestris* protects against insectivorous birds but not against predatory lacewing larvae (Petschenka et al., 2022; Pokharel et al., 2020). The predatory effectiveness of insect-sequestered toxins depends strongly on the chemistry of the host plant (Pokharel et al., 2020). The deterrence potential of different toxins from various plant species with a range of different amounts and diversity against predators (both invertebrates and vertebrates) should be investigated to improve our understanding of the ecological implications of sequestered PSMs. Nevertheless, the evolution of chemical defences is linked to trade-offs between the acquired benefits (through protection) and the costs of acquiring chemicals. Since chemical defences directly increase the survival of well-defended prey against predators, ecological costs are not easily apparent.

## Conclusions

No-cost meals might not exist for insects that feed on toxic plants. Theoretically, the costs of chemical defence can be estimated by examining at the tissue/cellular level (i.e. each step of toxin detoxification/sequestration) and organism level (i.e. the universality of defence) (Fig. 3). However, it is difficult to measure empirically because various traits of an organism interact depending upon physiological conditions (e.g. age and fecundity) and ecological dynamics (e.g. foraging and predation). The trade-offs may only appear in a species (or even within the same species) if a specific set of circumstances is met (Reznick, 1985). However, we can attempt to estimate using a manipulative and reductionist approach. For the most accurate and revealing information, one should think about using a single-factor manipulation (Stearns, 1992). In agricultural systems, research on the costs of pesticide resistance will help us better understand how evolution works and will provide useful data for pest management (Box 2).

**Box 2. Costs of resistance to insecticides**

Over a century has passed with records of insecticide resistance (Melander, 1914). Numerous investigations have been conducted on this fascinating evolutionary process, revealing its molecular underpinnings and effects on multiple arthropods (Feyereisen, 1995). Insect populations often evolve resistance to insecticides, enabling insects to flourish in agroecosystems (Coustau et al., 2000). Nevertheless, physiological and biochemical changes are expected as a result of selection for insecticide resistance (Feyereisen et al., 2015). Resistance to pesticides is frequently linked to negative impacts on fitness (see examples here Kliot and Ghanim, 2012). However, several studies conducted on various insect species reveal that resistance to insecticides does not inevitably lead to fitness costs. For example, the fitness of the malathion resistant red flour beetle, *Tribolium castaneum*, was not affected and was independent of the genetic background (Haubruge and Arnaud, 2001). Interestingly, a single allele conferred a fitness advantage in the absence of selective insecticide in DDT-resistant fruit flies, *Drosophila melanogaster* (McCart et al., 2005). In diflubenzuron- and deltamethrin-resistant codling moths, *Cydia pomonella*, deleterious pleiotropy of resistance was observed wherein the fitness cost was mainly associated to metabolic resistance (Boivin et al., 2003). Pyrethroid-resistant populations of the maize weevil, *Sitophilus zeamais*, showed an energy trade-off (here, energy reserves, i.e. trophocyte area) between resistance and life history traits such as development and reproduction (Guedes et al., 2006). The discrepancy might be due to invisible costs under the studied conditions (i.e. environmental factors), or costs might not even exist depending on the resistance mechanism. Therefore, in addition to applied use in pest management programmes, research on insecticide resistance is significant as a model to understand the evolution of novel phenotypes and the change in physiological and genetic traits.

We recently reviewed methods to study the interaction between PSM and insect herbivores and recommend them for further reading (Jeckel et al., 2022). Briefly, transgenic approaches can be employed to create insect lines to measure the true costs of resistance and sequestration. Here, I review and suggest where costs could be detected in an ecophysiological framework. Both the aspects of chemical defence, the multifaceted effects on insect physiology (i.e. physiological costs) and the context-dependent effect against predators (i.e. ecological costs) are important for our better understanding of the ecology and evolution of plant-insect-antagonist interaction. I hope this Review will stimulate further interest in unanswered questions about the costs of chemical defences.

## References

[BIO059800C1] Abdelfattah, E. A. and El-Bassiony, G. M. (2022). Impact of malathion toxicity on the oxidative stress parameters of the black soldier fly Hermetia illucens (Linnaeus, 1758) (Diptera: Stratiomyidae). *Sci. Rep.* 12, 4583. 10.1038/s41598-022-08564-8 35301370 PMC8931003

[BIO059800C2] Abrams, P. A. (2000). The evolution of predator-prey interactions: theory and evidence. *Annu. Rev. Ecol. Syst.* 31, 79-105. 10.1146/annurev.ecolsys.31.1.79

[BIO059800C3] Adamo, S. A. (2004). How should behavioural ecologists interpret measurements of immunity? *Anim. Behav.* 6, 1443-1449. 10.1016/j.anbehav.2004.05.005

[BIO059800C4] Adams, K. L., Aljohani, A., Chavez, J. and de Roode, J. C. (2021). Effects of cardenolides of milkweed plants on immunity of the monarch butterfly. *Arthropod-Plant Int.* 15, 249-252. 10.1007/s11829-021-09812-w

[BIO059800C5] Agrawal, A. A., Böröczky, K., Haribal, M., Hastings, A. P., White, R. A., Jiang, R.-W. and Duplais, C. (2021). Cardenolides, toxicity, and the costs of sequestration in the coevolutionary interaction between monarchs and milkweeds. *Proc. Natl Acad. Sci. USA* 118, e2024463118. 10.1073/pnas.2024463118 33850021 PMC8072370

[BIO059800C6] Agrawal, A. A., Espinosa del Alba, L., López-Goldar, X., Hastings, A. P., White, R. A., Halitschke, R., Dobler, S., Petschenka, G. and Duplais, C. (2022). Functional evidence supports adaptive plant chemical defense along a geographical cline. *Proc. Natl Acad. Sci. USA* 119, e2205073119. 10.1073/pnas.2205073119 35696564 PMC9231628

[BIO059800C7] Aucoin, R. R., Fields, P., Lewis, M. A., Philogène, B. J. R. and Arnason, J. T. (1990). The protective effect of antioxidants to a phototoxin-sensitive insect herbivore,Manduca sexta. *J. Chem. Ecol.* 16, 2913-2924. 10.1007/BF00979483 24263264

[BIO059800C8] Aucoin, R. R., Philogène, B. J. R. and Arnason, J. T. (1991). Antioxidant enzymes as biochemical defenses against phototoxin-induced oxidative stress in three species of herbivorous lepidoptera. *Arch. Insect Biochem. Physiol.* 16, 139-152. 10.1002/arch.940160206

[BIO059800C9] Aucoin, R., Guillet, G., Murray, C., Philogène, B. J. R. and Arnason, J. T. (1995). How do insect herbivores cope with the extreme oxidative stress of phototoxic host plants? *Arch. Insect Biochem. Physiol.* 29, 211-226. 10.1002/arch.940290210 28833490

[BIO059800C10] Balaban, R. S., Nemoto, S. and Finkel, T. (2005). Mitochondria, oxidants, and aging. *Cell* 120, 483-495. 10.1016/j.cell.2005.02.001 15734681

[BIO059800C11] Barbehenn, R. V. and Stannard, J. (2004). Antioxidant defense of the midgut epithelium by the peritrophic envelope in caterpillars. *J. Insect Physiol.* 50, 783-790. 10.1016/j.jinsphys.2004.05.012 15350499

[BIO059800C12] Barthel, A., Vogel, H., Pauchet, Y., Pauls, G., Kunert, G., Groot, A. T., Boland, W., Heckel, D. G. and Heidel-Fischer, H. M. (2016). Immune modulation enables a specialist insect to benefit from antibacterial withanolides in its host plant. *Nat. Commun.* 7, 1-11. 10.1038/ncomms12530 PMC5007441 27561781

[BIO059800C13] Beckage, N. E. (2011). *Insect Immunology*. Academic Press.

[BIO059800C14] Beckman, K. B. and Ames, B. N. (1998). The free radical theory of aging matures. *Physiol. Rev.* 78, 547-581. 10.1152/physrev.1998.78.2.547 9562038

[BIO059800C15] Beran, F. and Petschenka, G. (2022). Sequestration of plant defense compounds by insects: from mechanisms to insect–plant coevolution. *Annu. Rev. Entomol.* 67, 163-180. 10.1146/annurev-ento-062821-062319 34995091

[BIO059800C16] Bergelson, J., Dwyer, G. and Emerson, J. (2001). Models and data on plant-enemy coevolution. *Annu. Rev. Genet.* 35, 469-499. 10.1146/annurev.genet.35.102401.090954 11700291

[BIO059800C17] Bhattacharya, S. (2015). *Free Radicals in Human Health and Disease* (ed. V. Rani and U. C. S. Yadav), pp. 17-29, New Delhi: Springer India.

[BIO059800C18] Bhattamisra, S. K., Yap, K. H., Rao, V. and Choudhury, H. (2020). Multiple biological effects of an iridoid glucoside, catalpol, and its underlying molecular mechanisms. *Biomolecules* 10, 32. 10.3390/biom10010032 PMC7023090 31878316

[BIO059800C19] Birnbaum, S. S. L., Rinker, D. C., Gerardo, N. M. and Abbot, P. (2017). Transcriptional profile and differential fitness in a specialist milkweed insect across host plants varying in toxicity. *Mol. Ecol.* 26, 6742-6761. 10.1111/mec.14401 29110382

[BIO059800C20] Blakley, N. and Goodner, S. R. (1978). Size-dependent timing of metamorphosis in milkweed bugs (Oncopeltus) and its life history implications. *Biol. Bull.* 155, 499-510. 10.2307/1540786

[BIO059800C21] Blount, J. D., Rowland, H. M., Mitchell, C., Speed, M. P., Ruxton, G. D., Endler, J. A. and Brower, L. P. (2023). The price of defence: toxins, visual signals and oxidative state in an aposematic butterfly. *Proc. R. Soc. B* 290, 20222068. 10.1098/rspb.2022.2068 PMC9845971 36651049

[BIO059800C22] Blum, M. (2012). *Chemical Defenses of Arthropods*. Elsevier.

[BIO059800C23] Boivin, T., Bouvier, J. C., Chadœuf, J., Beslay, D. and Sauphanor, B. (2003). Constraints on adaptive mutations in the codling moth Cydia pomonella (L.): measuring fitness trade-offs and natural selection. *Heredity* 90, 107-113. 10.1038/sj.hdy.6800188 12522433

[BIO059800C24] Bowers, M. D. (1992). The evolution of unpalatability and the cost of chemical defense in insects. In *Insect Chemical Ecology: An Evolutionary Approach* (ed. B. D. Roitberg and M. B. Isman), pp. 216-244. New York, NY, USA: Chapman & Hall. https://books.google.de/books?hl=en&lr=&id=p5oah9s-ld8C&oi=fnd&pg=PA216&dq=info:qMidxxnsjAcJ:scholar.google.com&ots=s5 wRK3s_fF&sig=x3WapPi36U7TXW4YqdL8B5nLspQ&redir_esc=y#v=one page &q&f=false

[BIO059800C25] Bowers, M. D. and Puttick, G. M. (1988). Response of generalist and specialist insects to qualitative allelochemical variation. *J. Chem. Ecol.* 14, 319-334. 10.1007/BF01022549 24277012

[BIO059800C26] Bowers, M. D. and Puttick, G. M. (1989). Iridoid glycosides and insect feeding preferences: gypsy moths (Lymantria dispar, Lymantriidae) and buckeyes (Junonia coenia, Nymphalidae). *Ecol. Entomol.* 14, 247-256.

[BIO059800C27] Bramer, C., Friedrich, F. and Dobler, S. (2017). Defence by plant toxins in milkweed bugs (Heteroptera: Lygaeinae) through the evolution of a sophisticated storage compartment. *Syst. Entomol.* 42, 15-30. 10.1111/syen.12189

[BIO059800C28] Brown, K. S. and Francini, R. B. (1990). Evolutionary strategies of chemical defense in aposematic butterflies: Cyanogenesis in Asteraceae-feeding American Acraeinae. *Chemoecology* 1, 52-56. 10.1007/BF01325228

[BIO059800C29] Bura, V. L., Kawahara, A. Y. and Yack, J. E. (2016). A comparative analysis of sonic defences in Bombycoidea caterpillars. *Sci. Rep.* 6, 31469. 10.1038/srep31469 27510510 PMC4980592

[BIO059800C30] Camara, M. D. (1997). Predator responses to sequestered plant toxins in buckeye caterpillars: are tritrophic interactions locally variable? *J. Chem. Ecol.* 23, 2093-2106. 10.1023/B:JOEC.0000006431.34359.c2

[BIO059800C31] Catania, K. C. (2018). How not to be turned into a zombie. *Brain Behav. Evol.* 92, 32-46. 10.1159/000490341 30380540 PMC6390464

[BIO059800C32] Celestino, D., Braoios, G. I., Ramos, R. S., Gontijo, L. M. and Guedes, R. N. C. (2014). Azadirachtin-mediated reproductive response of the predatory pirate bug Blaptostethus pallescens. *BioControl* 59, 697-705. 10.1007/s10526-014-9601-z

[BIO059800C33] Chahine, S. and O'Donnell, M. J. (2011). Interactions between detoxification mechanisms and excretion in Malpighian tubules of Drosophila melanogaster. *J. Exp. Biol.* 214, 462-468. 10.1242/jeb.048884 21228205

[BIO059800C34] Chaplin, S. J. and Chaplin, S. B. (1981). Growth dynamics of a specialized milkweed seed feeder (Oncopeltus fasciatus) on seeds of familiar and unfamiliar milkweeds (Asclepias spp.). *Entomol. Exp. Appl.* 29, 345-356. 10.1111/j.1570-7458.1981.tb03078.x

[BIO059800C35] Charles, H. M. and Killian, K. A. (2015). Response of the insect immune system to three different immune challenges. *J. Insect Physiol.* 81, 97-108. 10.1016/j.jinsphys.2015.07.005 26164746

[BIO059800C36] Clark, C. W. and Harvell, C. D. (1992). Inducible defenses and the allocation of resources: a minimal model. *Am. Nat.* 139, 521-539. 10.1086/285342

[BIO059800C37] Correa-Garhwal, S. M., Baker, R. H., Clarke, T. H., Ayoub, N. A. and Hayashi, C. Y. (2022). The evolutionary history of cribellate orb-weaver capture thread spidroins. *BMC Ecol. Evol.* 22, 89. 10.1186/s12862-022-02042-5 35810286 PMC9270836

[BIO059800C38] Costantini, D. (2008). Oxidative stress in ecology and evolution: lessons from avian studies. *Ecol. Lett.* 11, 1238-1251. 10.1111/j.1461-0248.2008.01246.x 18803642

[BIO059800C39] Costantini, D. (2019). Understanding diversity in oxidative status and oxidative stress: the opportunities and challenges ahead. *J. Exp. Biol.* 222, jeb194688. 10.1242/jeb.194688 31266782

[BIO059800C40] Coustau, C., Chevillon, C. and ffrench-Constant, R. (2000). Resistance to xenobiotics and parasites: can we count the cost? *Trends Ecol. Evol.* 15, 378-383. 10.1016/S0169-5347(00)01929-7 10931680

[BIO059800C41] Dobler, S., Dalla, S., Wagschal, V. and Agrawal, A. A. (2012). Community-wide convergent evolution in insect adaptation to toxic cardenolides by substitutions in the Na,K-ATPase. *Proc. Natl. Acad. Sci. USA* 109, 13040. 10.1073/pnas.1202111109 22826239 PMC3420205

[BIO059800C42] Dowling, D. K. and Simmons, L. W. (2009). Reactive oxygen species as universal constraints in life-history evolution. *Proc. R. Soc. B* 276, 1737-1745. 10.1098/rspb.2008.1791 PMC2674489 19324792

[BIO059800C43] Eisner, T., Eisner, M. and Siegler, M. (2005). *Secret Weapons: Defenses of Insects, Spiders, Scorpions and Other Many-Legged Creatures*. Cambridge: Belknap Press of Harvard University Press.

[BIO059800C44] Enayati, A. A., Ranson, H. and Hemingway, J. (2005). Insect glutathione transferases and insecticide resistance. *Insect Mol. Biol.* 14, 3-8. 10.1111/j.1365-2583.2004.00529.x 15663770

[BIO059800C45] Erickson, J. M. (1973). The utilization of various Asclepias species by larvae of the monarch butterfly, Danaus plexippus. *Psyche* 80, 230-244. 10.1155/1973/28693

[BIO059800C46] Feyereisen, R. (1995). Molecular biology of insecticide resistance. *Toxicol. Lett.* 82-83, 83-90. 10.1016/0378-4274(95)03470-6 8597150

[BIO059800C47] Feyereisen, R., Dermauw, W. and Van Leeuwen, T. (2015). Genotype to phenotype, the molecular and physiological dimensions of resistance in arthropods. *Pestic. Biochem. Physiol.* 121, 61-77. 10.1016/j.pestbp.2015.01.004 26047113

[BIO059800C48] Finkel, T. and Holbrook, N. J. (2000). Oxidants, oxidative stress and the biology of ageing. *Nature* 408, 239-247. 10.1038/35041687 11089981

[BIO059800C49] Fry, J. D. (2003). Detecting ecological trade–offs using selection experiments. *Ecology* 84, 1672-1678. 10.1890/0012-9658(2003)084[1672:DETUSE]2.0.CO;2

[BIO059800C50] Gentry, G. L. and Dyer, L. A. (2002). On the conditional nature of neotropical caterpillar defenses against their natural enemies. *Ecology* 83, 3108-3119. 10.1890/0012-9658(2002)083[3108:OTCNON]2.0.CO;2

[BIO059800C51] Gnatzy, W. and Heusslein, R. (1986). Digger wasp against crickets. *Naturwissenschaften* 73, 212-215. 10.1007/BF00417728

[BIO059800C52] Gowler, C. D., Leon, K. E., Hunter, M. D. and de Roode, J. C. (2015). Secondary defense chemicals in milkweed reduce parasite infection in monarch butterflies, Danaus plexippus. *J. Chem. Ecol.* 41, 520-523. 10.1007/s10886-015-0586-6 25953502

[BIO059800C53] Guedes, R. N. C., Oliveira, E. E., Guedes, N. M. P., Ribeiro, B. and Serrão, J. E. (2006). Cost and mitigation of insecticide resistance in the maize weevil, Sitophilus zeamais. *Physiol. Entomol.* 31, 30-38. 10.1111/j.1365-3032.2005.00479.x

[BIO059800C54] Hague, M. T. J., Toledo, G., Geffeney, S. L., Hanifin, C. T., Brodie, E. D., Jr and Brodie, E. D. III. (2018). Large-effect mutations generate trade-off between predatory and locomotor ability during arms race coevolution with deadly prey. *Evol. Lett.* 2, 406-416. 10.1002/evl3.76 30283691 PMC6121790

[BIO059800C55] Hamama, H. M. and Fergani, Y. A. (2019). Toxicity and oxidative stress induced in *Spodoptera littoralis* (Boisduval) (Lepidoptera : Noctuidae) treated with some insecticides. *Afr. Entomol.* 27, 523-531. 10.4001/003.027.0523

[BIO059800C56] Hanifin, C. T. (2010). The chemical and evolutionary ecology of tetrodotoxin (TTX) toxicity in terrestrial vertebrates. *Mar. Drugs* 8, 577-593. 10.3390/md8030577 20411116 PMC2857372

[BIO059800C57] Hartmann, T. (2004). Plant-derived secondary metabolites as defensive chemicals in herbivorous insects: a case study in chemical ecology. *Planta* 219, 1-4. 10.1007/s00425-004-1249-y 15042370

[BIO059800C58] Harvey, J. A., Van Nouhuys, S. and Biere, A. (2005). Effects of quantitative variation in allelochemicals in Plantago lanceolata on development of a generalist and a specialist herbivore and their endoparasitoids. *J. Chem. Ecol.* 31, 287-302. 10.1007/s10886-005-1341-1 15856784

[BIO059800C59] Haubruge, E. and Arnaud, L. (2001). Fitness consequences of malathion-specific resistance in red flour beetle (Coleoptera: Tenebrionidae) and selection for resistance in the absence of malathion. *J. Econ. Entomol.* 94, 552-557. 10.1603/0022-0493-94.2.552 11332853

[BIO059800C60] Heckel, D. G. (2014). Insect detoxification and sequestration strategies. *Ann. Plant Rev.* 47, 77-114. 10.1002/9781118829783.ch3

[BIO059800C61] Heyworth, H. C., Pokharel, P., Blount, J. D., Mitchell, C., Petschenka, G. and Rowland, H. M. (2023). Antioxidant availability trades off with warning signals and toxin sequestration in the large milkweed bug (Oncopeltus fasciatus). *Ecol. Evol.* 13, e9971. 10.1002/ece3.9971 37038513 PMC10082154

[BIO059800C62] Houston, A. I., McNamara, J. M. and Hutchinson, J. M. (1993). General results concerning the trade-off between gaining energy and avoiding predation. *Phil. Trans. R. Soc. Lond. B Biol. Sci.* 341, 375-397. 10.1098/rstb.1993.0123

[BIO059800C63] Humphreys, R. K. and Ruxton, G. D. (2018). A review of thanatosis (death feigning) as an anti-predator behaviour. *Behav. Ecol. Sociobiol.* 72, 22. 10.1007/s00265-017-2436-8 29386702 PMC5769822

[BIO059800C64] Isaksson, C., Sheldon, B. C. and Uller, T. (2011). The Challenges of Integrating Oxidative Stress into Life-history Biology. *Bioscience* 61, 194-202. 10.1525/bio.2011.61.3.5

[BIO059800C65] Isman, M. B. (1977). Dietary influence of cardenolides on larval growth and development of the milkweed bug Oncopeltus fasciatus. *J. Insect Physiol.* 23, 1183-1187. 10.1016/0022-1910(77)90151-2

[BIO059800C66] Ito, F., Taniguchi, K. and Billen, J. (2016). Defensive function of petiole spines in queens and workers of the formicine ant Polyrhachis lamellidens (Hymenoptera: Formicidae) against an ant predator, the Japanese treefrog Hyla japonica. *Asian Myrmecol.* 8, 81-86. 10.20362/am.008014

[BIO059800C67] Jeckel, A. M., Beran, F., Züst, T., Younkin, G., Petschenka, G., Pokharel, P., Dreisbach, D., Ganal-Vonarburg, S. C. and Robert, C. A. M. (2022). Metabolization and sequestration of plant specialized metabolites in insect herbivores: Current and emerging approaches. *Front. Physiol.* 13, 1001032. 10.3389/fphys.2022.1001032 36237530 PMC9552321

[BIO059800C68] Kafel, A., Babczyńska, A., Zawisza-Raszka, A., Tarnawska, M., Płachetka-Bożek, A. and Augustyniak, M. (2021). Energy reserves, oxidative stress and development traits of Spodoptera exigua Hübner individuals from cadmium strain. *Environ. Pollut.* 268, 115366. 10.1016/j.envpol.2020.115366 33035914

[BIO059800C69] Karageorgi, M., Groen, S. C., Sumbul, F., Pelaez, J. N., Verster, K. I., Aguilar, J. M., Hastings, A. P., Bernstein, S. L., Matsunaga, T., Astourian, M. et al. (2019). Genome editing retraces the evolution of toxin resistance in the monarch butterfly. *Nature* 574, 409-412. 10.1038/s41586-019-1610-8 31578524 PMC7039281

[BIO059800C70] Kliot, A. and Ghanim, M. (2012). Fitness costs associated with insecticide resistance. *Pest Manag. Sci.* 68, 1431-1437. 10.1002/ps.3395 22945853

[BIO059800C71] Koricheva, J. (2002). Meta–analysis of sources of variation in fitness costs of plant antiherbivore defenses. *Ecology* 83, 176-190. 10.1890/0012-9658(2002)083[0176:MAOSOV]2.0.CO;2

[BIO059800C72] Kowalski, P., Baum, M., Körten, M., Donath, A. and Dobler, S. (2020). ABCB transporters in a leaf beetle respond to sequestered plant toxins. *Proc. R. Soc. B* 287, 20201311. 10.1098/rspb.2020.1311 PMC7542790 32873204

[BIO059800C73] Krishnan, N. and Sehnal, F. (2006). Compartmentalization of oxidative stress and antioxidant defense in the larval gut of Spodoptera littoralis. *Arch. Insect Biochem. Physiol.* 63, 1-10. 10.1002/arch.20135 16921519

[BIO059800C74] Krueger, A. J., Robinson, E. A., Weissling, T. J., Vélez, A. M. and Anderson, T. D. (2021). Cardenolide, potassium, and pyrethroid insecticide combinations reduce growth and survival of monarch butterfly caterpillars (Lepidoptera: Nymphalidae). *J. Econ. Entomol.* 114, 2370-2380. 10.1093/jee/toab169 34532742

[BIO059800C75] Kuraishi, T., Binggeli, O., Opota, O., Buchon, N. and Lemaitre, B. (2011). Genetic evidence for a protective role of the peritrophic matrix against intestinal bacterial infection in Drosophila melanogaster. *Proc. Natl Acad. Sci. USA* 108, 15966-15971. 10.1073/pnas.1105994108 21896728 PMC3179054

[BIO059800C76] Lampert, E. C. (2020). Relationships among catalpol sequestration, metabolism and nutritional efficiencies of the catalpa sphinx, Ceratomia catalpae (Lepidoptera: Sphingidae). *Entomol. Sci.* 23, 196-203. 10.1111/ens.12413

[BIO059800C77] Lampert, E. (2012). Influences of plant traits on immune responses of specialist and generalist herbivores. *Insects* 3, 573-592. 10.3390/insects3020573 26466545 PMC4553612

[BIO059800C78] Laurentz, M., Reudler, J. H., Mappes, J., Friman, V., Ikonen, S. and Lindstedt, C. (2012). Diet quality can play a critical role in defense efficacy against parasitoids and pathogens in the Glanville fritillary (Melitaea cinxia). *J. Chem. Ecol.* 38, 116-125. 10.1007/s10886-012-0066-1 22273742

[BIO059800C79] Li, M., Fry, B. G. and Kini, R. M. (2005). Eggs-Only Diet: Its Implications for the Toxin Profile Changes and Ecology of the Marbled Sea Snake (Aipysurus eydouxii). *J. Mol. Evol.* 60, 81-89. 10.1007/s00239-004-0138-0 15696370

[BIO059800C80] Lindstedt, C., Talsma, J. H. R., Ihalainen, E., Lindström, L. and Mappes, J. (2010). Diet quality affects warning coloration indirectly: excretion costs in a generalist herbivore. *Evolution* 64, 68-78. 10.1111/j.1558-5646.2009.00796.x 19659593

[BIO059800C81] Liu, S., Niu, H., Xiao, T., Xue, C., Liu, Z. and Luo, W. (2009). Does phenoloxidase contributed to the resistance? Selection with butane-fipronil enhanced its activities from diamondback moths. *Open Biochem. J.* 3, 9. 10.2174/1874091X00903010009 19401784 PMC2674291

[BIO059800C82] Mailho-Fontana, P. L., Jared, C., Antoniazzi, M. M., Sciani, J. M., Pimenta, D. C., Stokes, A. N., Grant, T., Brodie, E. D. and Brodie, E. D. (2019). Variations in tetrodotoxin levels in populations of Taricha granulosa are expressed in the morphology of their cutaneous glands. *Sci. Rep.* 9, 18490. 10.1038/s41598-019-54765-z 31811169 PMC6897900

[BIO059800C83] Mappes, J., Marples, N. and Endler, J. A. (2005). The complex business of survival by aposematism. *Trends Ecol. Evol.* 20, 598-603. 10.1016/j.tree.2005.07.011 16701442

[BIO059800C84] McCart, C., Buckling, A. and ffrench-Constant, R. H. (2005). DDT resistance in flies carries no cost. *Curr. Biol.* 15, R587-R589. 10.1016/j.cub.2005.07.054 16085476

[BIO059800C85] McCue, M. D. (2006). Cost of producing venom in three north american pitviper species. *Copeia* 2006, 818-825. 10.1643/0045-8511(2006)6[818:COPVIT]2.0.CO;2

[BIO059800C86] McGraw, K. J. (2005). The antioxidant function of many animal pigments: are there consistent health benefits of sexually selected colourants? *Anim. Behav.* 69, 757-764. 10.1016/j.anbehav.2004.06.022

[BIO059800C87] McMillan, L. E., Miller, D. W. and Adamo, S. A. (2018). Eating when ill is risky: immune defense impairs food detoxification in the caterpillar Manduca sexta. *J. Exp. Biol.* 221, jeb173336. 10.1242/jeb.173336 29217626

[BIO059800C88] McPeek, M. A. (2004). The growth/predation risk trade-off: so what is the mechanism? *Am. Nat.* 163, E88-E111. 10.1086/382755 15122497

[BIO059800C89] Melander, A. L. (1914). Can insects become resistant to sprays?1. *J. Econ. Entomol.* 7, 167-173. 10.1093/jee/7.2.167

[BIO059800C90] Metcalfe, N. B. and Alonso-Alvarez, C. (2010). Oxidative stress as a life-history constraint: the role of reactive oxygen species in shaping phenotypes from conception to death. *Funct. Ecol.* 24, 984-996. 10.1111/j.1365-2435.2010.01750.x

[BIO059800C91] Monaghan, P., Metcalfe, N. B. and Torres, R. (2009). Oxidative stress as a mediator of life history trade-offs: mechanisms, measurements and interpretation. *Ecol. Lett.* 12, 75-92. 10.1111/j.1461-0248.2008.01258.x 19016828

[BIO059800C92] Muchoney, N. D., Bowers, M. D., Carper, A. L., Mason, P. A., Teglas, M. B. and Smilanich, A. M. (2022). Use of an exotic host plant shifts immunity, chemical defense, and viral burden in wild populations of a specialist insect herbivore. *Ecol. Evol.* 12, e8723. 10.1002/ece3.8723 35342612 PMC8928866

[BIO059800C93] Murphy, S. M., Leahy, S. M., Williams, L. S. and Lill, J. T. (2009). Stinging spines protect slug caterpillars (Limacodidae) from multiple generalist predators. *Behav. Ecol.* 21, 153-160. 10.1093/beheco/arp166

[BIO059800C94] Musser, R. O., Hum-Musser, S. M., Eichenseer, H., Peiffer, M., Ervin, G., Murphy, J. B. and Felton, G. W. (2002). Caterpillar saliva beats plant defences. *Nature* 416, 599-600. 10.1038/416599a 11948341

[BIO059800C95] Neilson, E. H., Goodger, J. Q., Woodrow, I. E. and Møller, B. L. (2013). Plant chemical defense: at what cost? *Trends Plant Sci.* 18, 250-258. 10.1016/j.tplants.2013.01.001 23415056

[BIO059800C96] Nguyen, K.-O., Al-Rashid, S., Clarke Miller, M., Tom Diggs, J. and Lampert, E. C. (2019). Trichoplusia ni (Lepidoptera: Noctuidae) qualitative and quantitative sequestration of host plant carotenoids. *Environ. Entomol.* 48, 540-545. 10.1093/ee/nvz029 30951592

[BIO059800C97] Nisani, Z., Dunbar, S. G. and Hayes, W. K. (2007). Cost of venom regeneration in Parabuthus transvaalicus (Arachnida: Buthidae). *Comp. Biochem. Physiol. A Mol. Integr. Physiol.* 147, 509-513. 10.1016/j.cbpa.2007.01.027 17344080

[BIO059800C98] Nishida, R. (2002). Sequestration of defensive substances from plants by lepidoptera. *Annu. Rev. Entomol.* 47, 57-92. 10.1146/annurev.ento.47.091201.145121 11729069

[BIO059800C99] Opitz, S. E. W. and Müller, C. (2009). Plant chemistry and insect sequestration. *Chemoecology* 19, 117. 10.1007/s00049-009-0018-6

[BIO059800C100] Oron, U. and Bdolah, A. (1973). Regulation of protein synthesis in the venom gland of viperid snakes. *J. Cell Biol.* 56, 177-190. 10.1083/jcb.56.1.177 4345163 PMC2108839

[BIO059800C101] Palma Onetto, V., Oliva, D. and González Teuber, M. (2021). Lethal and oxidative stress side effects of organic and synthetic pesticides on the insect scale predator Rhyzobius lophanthae. *Entomol. Gen.* 41, 345-355.

[BIO059800C102] Pasteels, J. M., Braekman, J.-C. and Daloze, D. (1988). Biology of Chrysomelidae (ed. P. Jolivet, E. Petitpierre and T. H. Hsiao), pp. 233-252. Dordrecht: Springer Netherlands.

[BIO059800C103] Perret, B. A. (1977). Proteolytic activity of tarantula venoms due to contamination with saliva. *Toxicon* 15, 505-510. 10.1016/0041-0101(77)90101-5 906036

[BIO059800C104] Perveen, N. and Ahmad, M. (2017). Toxicity of some insecticides to the haemocytes of giant honeybee, Apis dorsata F. under laboratory conditions. *Saudi J. Biol. Sci.* 24, 1016-1022. 10.1016/j.sjbs.2016.12.011 28663697 PMC5478291

[BIO059800C105] Petersen, R. A., Zangerl, A. R., Berenbaum, M. R. and Schuler, M. A. (2001). Expression of CYP6B1 and CYP6B3 cytochrome P450 monooxygenases and furanocoumarin metabolism in different tissues of Papilio polyxenes (Lepidoptera: Papilionidae). *Insect Biochem. Mol. Biol.* 31, 679-690. 10.1016/S0965-1748(00)00174-0 11267906

[BIO059800C106] Petschenka, G. and Agrawal, A. A. (2015). Milkweed butterfly resistance to plant toxins is linked to sequestration, not coping with a toxic diet. *Proc. R. Soc. B* 282, 20151865. 10.1098/rspb.2015.1865 PMC4650158 26538594

[BIO059800C107] Petschenka, G. and Agrawal, A. A. (2016). How herbivores coopt plant defenses: natural selection, specialization, and sequestration. *Current Opinion in Insect Science* 14, 17-24. 10.1016/j.cois.2015.12.004 27436642

[BIO059800C108] Petschenka, G., Halitschke, R., Züst, T., Roth, A., Stiehler, S., Tenbusch, L., Hartwig, C., Gámez, J. F. M., Trusch, R., Deckert, J. et al. (2022). Sequestration of defenses against predators drives specialized host plant associations in preadapted milkweed bugs (Heteroptera: Lygaeinae). *Am. Nat.* 199, E211-E228. 10.1086/719196 35580225

[BIO059800C109] Piiroinen, S., Boman, S., Lyytinen, A., Mappes, J. and Lindström, L. (2014). Sublethal effects of deltamethrin exposure of parental generations on physiological traits and overwintering in L eptinotarsa decemlineata. *J. Appl. Entomol.* 138, 149-158. 10.1111/jen.12088

[BIO059800C110] Pocius, V. M., Cibotti, S., Ray, S., Ankoma-Darko, O., McCartney, N. B., Schilder, R. J. and Ali, J. G. (2022). Impacts of larval host plant species on dispersal traits and free-flight energetics of adult butterflies. *Commun. Biol.* 5, 469. 10.1038/s42003-022-03396-8 35577926 PMC9110344

[BIO059800C111] Pokharel, P. (2023). Physiological and ecological implications of sequestered cardenolides in the milkweed bugs (Heteroptera: Lygaeinae). *PhD Thesis*, Kommunikations-, Informations- und Medienzentrum der Universität Hohenheim, Stuttgart, Germany.

[BIO059800C112] Pokharel, P., Sippel, M., Vilcinskas, A. and Petschenka, G. (2020). Defense of milkweed bugs (Heteroptera: Lygaeinae) against predatory lacewing larvae depends on structural differences of sequestered cardenolides. *Insects* 11, 485. 10.3390/insects11080485 32752003 PMC7469174

[BIO059800C113] Pokharel, P., Steppuhn, A. and Petschenka, G. (2021). Dietary cardenolides enhance growth and change the direction of the fecundity-longevity trade-off in milkweed bugs (Heteroptera: Lygaeinae). *Ecol. Evol.* 11, 18042-18054. 10.1002/ece3.8402 35003656 PMC8717354

[BIO059800C114] Rahman, M. M., Roberts, H. L., Sarjan, M., Asgari, S. and Schmidt, O. (2004). Induction and transmission of Bacillus thuringiensis tolerance in the flour moth Ephestia kuehniella. *Proc. Natl Acad. Sci. USA* 101, 2696-2699. 10.1073/pnas.0306669101 14978282 PMC365683

[BIO059800C115] Ratzka, A., Vogel, H., Kliebenstein, D. J., Mitchell-Olds, T. and Kroymann, J. (2002). Disarming the mustard oil bomb. *Proc. Natl Acad. Sci. USA* 99, 11223-11228. 10.1073/pnas.172112899 12161563 PMC123237

[BIO059800C116] Renault, D., Dorrah, M. A., Mohamed, A. A., Abdelfattah, E. A. and Bassal, T. T. M. (2016). Assessment of oxidative stress and activities of antioxidant enzymes depicts the negative systemic effect of iron-containing fertilizers and plant phenolic compounds in the desert locust. *Envir. Sci. Pollut. Res.* 23, 21989-22000. 10.1007/s11356-016-7391-9 27539469

[BIO059800C117] Renwick, J. A. A. and Lopez, K. (1999). *Proceedings of the 10th International Symposium on Insect-Plant Relationships* (ed. S. J. Simpson, A. J. Mordue and J. Hardie), pp. 51-58. Dordrecht: Springer Netherlands.

[BIO059800C118] Reudler, J. H., Biere, A., Harvey, J. A. and Van Nouhuys, S. (2011). Differential performance of a specialist and two generalist herbivores and their parasitoids on Plantago lanceolata. *J. Chem. Ecol.* 37, 765-778. 10.1007/s10886-011-9983-7 21691810 PMC3125503

[BIO059800C119] Reynolds, C. J., Turin, D. R. and Romero, M. F. (2021). Transporters and tubule crystals in the insect Malpighian tubule. *Curr. Opin. Insect Sci.* 47, 82-89. 10.1016/j.cois.2021.05.003 34044181 PMC8487917

[BIO059800C120] Reznick, D. (1985). Costs of reproduction: an evaluation of the empirical evidence. *Oikos* 44, 257-267. 10.2307/3544698

[BIO059800C121] Roff, D. (1992). *The Evolution of Life Histories: Theory and Analysis*. New York: Chapman and Hall, 535.

[BIO059800C122] Rotenberg, D., Bamberger, E. S. and Kochva, E. (1971). Studies on ribonucleic acid synthesis in the venom glands of Vipera palaestinae (Ophidia, Reptilia). *Biochem. J.* 121, 609-612. 10.1042/bj1210609 4940047 PMC1176636

[BIO059800C123] Rothschild, M. L., Euw, J. V., Reichstein, T., Smith, D. and Pierre, J. (1975). Cardenolide storage in Danaus chrysippus (L.) with additional notes on D. plexippus (L.). *Proc. R. Soc. Lond. B Biol. Sci.* 190, 1-31.

[BIO059800C124] Rothschild, M., Aplin, R. T., Cockrum, P. A., Edgar, J. A., Fairweather, P. and Lees, R. (1979). Pyrrolizidine alkaloids in arctiid moths (Lep.) with a discussion on host plant relationships and the role of these secondary plant substances in the Arctiidae. *Biol. J. Linn. Soc.* 12, 305-326. 10.1111/j.1095-8312.1979.tb00062.x

[BIO059800C125] Ruxton, G. (2014). Empirically exploring why latex might be white: a comment on Lev-Yadun 2014. *Chemoecology* 24, 219-220. 10.1007/s00049-014-0162-5

[BIO059800C126] Ruxton, G. D., Allen, W. L., Sherratt, T. N. and Speed, M. P. (2019). *Avoiding Attack: the Evolutionary Ecology of Crypsis, Aposematism, And Mimicry*. Oxford University Press.

[BIO059800C127] Saastamoinen, M., van Nouhuys, S., Nieminen, M., O'Hara, B. and Suomi, J. (2007). Development and survival of a specialist herbivore, Melitaea cinxia, on host plants producing high and low concentrations of iridoid glycosides, pp. 70–80, JSTOR.

[BIO059800C128] Schmidt, J. O. (2016). *The Sting of the Wild*. JHU Press.

[BIO059800C129] Scudder, G. and Meredith, J. (1982). Morphological basis of cardiac glycoside sequestration by Oncopeltus fasciatus (Dallas)(Hemiptera: Lygaeidae). *Zoomorphology* 99, 87-101. 10.1007/BF00310302

[BIO059800C130] Sebastiano, M., Messina, S., Marasco, V. and Costantini, D. (2022). Hormesis in ecotoxicological studies: a critical evolutionary perspective. *Current Opinion in Toxicology* 29, 25-30. 10.1016/j.cotox.2022.01.002

[BIO059800C131] Shamim, G., Rajan, K. S., Pandey, M. D. and Ramani, R. (2014). Biochemistry and biosynthesis of insect pigments. *EJE* 111, 149-164.

[BIO059800C132] Smagghe, G. and Degheele, D. (1997). Comparative Toxicity and Tolerance for the Ecdysteroid Mimic Tebufenozide in a Laboratory and Field Strain of Cotton Leafworm (Lepidoptera: Noctuidae). *J. Econ. Entomol.* 90, 278-282. 10.1093/jee/90.2.278

[BIO059800C133] Smilanich, A. M. and Muchoney, N. D. (2022). *Caterpillars in the Middle*, pp. 449-484. Springer.

[BIO059800C134] Smilanich, A. M., Dyer, L. A., Chambers, J. Q. and Bowers, M. D. (2009). Immunological cost of chemical defence and the evolution of herbivore diet breadth. *Ecol. Lett.* 12, 612-621. 10.1111/j.1461-0248.2009.01309.x 19392713

[BIO059800C135] Smilanich, A. M., Vargas, J., Dyer, L. A. and Bowers, M. D. (2011). Effects of ingested secondary metabolites on the immune response of a polyphagous caterpillar Grammia incorrupta. *J. Chem. Ecol.* 37, 239-245. 10.1007/s10886-011-9924-5 21360275

[BIO059800C136] Speakman, J. R. and Garratt, M. (2014). Oxidative stress as a cost of reproduction: Beyond the simplistic trade-off model. *BioEssays* 36, 93-106. 10.1002/bies.201300108 24285005

[BIO059800C137] Stearns, S. C. (1992). The evolution of life histories.

[BIO059800C138] Steiner, U. K. and Pfeiffer, T. (2007). Optimizing time and resource allocation trade-offs for investment into morphological and behavioral defense. *Am. Nat.* 169, 118-129. 10.1086/509939 17206590

[BIO059800C139] Sternberg, E. D., Lefèvre, T., Li, J., de Castillejo, C. L. F., Li, H., Hunter, M. D. and de Roode, J. C. (2012). Food plant derived disease tolerance and resistance in a natural butterfly–plant–parasite interactions. *Evolution* 66, 3367-3376. 10.1111/j.1558-5646.2012.01693.x 23106703

[BIO059800C140] Stoks, R. (2001). Food stress and predator-induced stress shape developmental performance in a damselfly. *Oecologia* 127, 222-229. 10.1007/s004420000595 24577653

[BIO059800C141] Strauss, A. S., Peters, S., Boland, W. and Burse, A. (2013). ABC transporter functions as a pacemaker for sequestration of plant glucosides in leaf beetles. *eLife* 2, e01096. 10.7554/eLife.01096 24302568 PMC3843118

[BIO059800C142] Sugiura, S. and Yamazaki, K. (2014). Caterpillar hair as a physical barrier against invertebrate predators. *Behav. Ecol.* 25, 975-983. 10.1093/beheco/aru080

[BIO059800C143] Summers, C. B. and Felton, G. W. (1996). Peritrophic envelope as a functional antioxidant. *Arch. Insect Biochem. Physiol.* 32, 131-142. 10.1002/(SICI)1520-6327(1996)32:1<131::AID-ARCH8>3.0.CO;2-2

[BIO059800C144] Tan, W.-H., Tao, L., Hoang, K. M., Hunter, M. D. and de Roode, J. C. (2018). The effects of milkweed induced defense on parasite resistance in monarch butterflies, Danaus plexippus. *J. Chem. Ecol.* 44, 1040-1044. 10.1007/s10886-018-1007-4 30123937

[BIO059800C145] Tan, W. H., Acevedo, T., Harris, E. V., Alcaide, T. Y., Walters, J. R., Hunter, M. D., Gerardo, N. M. and de Roode, J. C. (2019). Transcriptomics of monarch butterflies (Danaus plexippus) reveals that toxic host plants alter expression of detoxification genes and down–regulate a small number of immune genes. *Mol. Ecol.* 28, 4845-4863. 10.1111/mec.15219 31483077

[BIO059800C146] Tan, K., Zhang, H., Lim, L.-S., Ma, H., Li, S. and Zheng, H. (2020). Roles of carotenoids in invertebrate immunology. *Front. Immunol.* 10, 3041. 10.3389/fimmu.2019.03041 32010132 PMC6979042

[BIO059800C147] Thompson, J. N. (2005). *The Geographic Mosaic of Coevolution*. University of Chicago Press.

[BIO059800C148] Veal, E. and Day, A. (2011). Hydrogen peroxide as a signaling molecule. *Antioxid Redox Signal.* 15, 147-151. 10.1089/ars.2011.3968 21375475

[BIO059800C149] Wink, M. (2009). Mode of action and toxicology of plant toxins and poisonous plants. *Mitt. Julius Kühn-Inst* 421, 93-112.

[BIO059800C150] Wink, M. (2018). Plant secondary metabolites modulate insect behavior-steps toward addiction? *Front. Physiol.* 9, 364. 10.3389/fphys.2018.00364 29695974 PMC5904355

[BIO059800C151] Yang, Z.-L., Nour-Eldin, H. H., Hänniger, S., Reichelt, M., Crocoll, C., Seitz, F., Vogel, H. and Beran, F. (2021). Sugar transporters enable a leaf beetle to accumulate plant defense compounds. *Nat. Commun.* 12, 2658. 10.1038/s41467-021-22982-8 33976202 PMC8113468

[BIO059800C152] Zagrobelny, M., Bak, S. and Møller, B. L. (2008). Cyanogenesis in plants and arthropods. *Phytochemistry* 69, 1457-1468. 10.1016/j.phytochem.2008.02.019 18353406

[BIO059800C153] Zhen, Y., Aardema, M. L., Medina, E. M., Schumer, M. and Andolfatto, P. (2012). Parallel molecular evolution in an herbivore community. *Science* 337, 1634. 10.1126/science.1226630 23019645 PMC3770729

[BIO059800C154] Zvereva, E. L. and Kozlov, M. V. (2016). The costs and effectiveness of chemical defenses in herbivorous insects: a meta–analysis. *Ecol. Monogr.* 86, 107-124.

